# Congenital cholesteatoma of the infratemporal fossa with congenital aural atresia and mastoiditis: a case report

**DOI:** 10.1186/1472-6815-12-6

**Published:** 2012-06-25

**Authors:** Mosaad Abdel-Aziz

**Affiliations:** 1 Department of Otolaryngology, Faculty of Medicine, Cairo University, Cairo, Egypt

**Keywords:** Congenital cholesteatoma, Congenital aural atresia, Mastoiditis, Infratemporal fossa

## Abstract

**Background:**

Congenital cholesteatoma may be expected in abnormally developed ear, it may cause bony erosion of the middle ear cleft and extend to the infratemporal fossa. We present the first case of congenital cholesteatoma of the infratemporal fossa in a patient with congenital aural atresia that has been complicated with acute mastoiditis.

**Case presentation:**

A sixteen year old Egyptian male patient presented with congenital cholesteatoma of the infratemporal fossa with congenital aural atresia complicated with acute mastoiditis. Two weeks earlier, the patient suffered pain necessitating hospital admission, magnetic resonance imaging revealed a soft tissue mass in the right infratemporal fossa. On presentation to our institute, Computerized tomography was done as a routine, it proved the diagnosis of mastoiditis, pure tone audiometry showed an air-bone gap of 60 dB. Cortical mastoidectomy was done for treatment of mastoiditis, removal of congenital cholesteatoma was carried out with reconstruction of external auditory canal. Follow-up of the patient for 2 years and 3 months showed a patent, infection free external auditory canal with an air-bone gap has been reduced to 35db. One year after the operation; MRI was done and it showed no residual or recurrent cholesteatoma.

**Conclusions:**

Congenital cholesteatoma of the infratemporal fossa in cases of congenital aural atresia can be managed safely even if it was associated with mastoiditis. It is an original case report of interest to the speciality of otolaryngology.

## Background

Congenital cholesteatoma (CC) is thought to occur secondary to failure of normal involution of epidermoid formation within the middle ear cleft. A collection of stratified squamous epidermoid cells appears during embryonic development and are instrumental in the development of middle ear mucosa. These remaining squamous cells grow slowly and become clinically apparent as a cholesteatoma usually during childhood [1]. However, CC may develop in various temporal bone sites, including the petrous apex, cerebellopontine angle, middle-ear cavity and mastoid process [2].

Although it is rare, the incidence of congenital cholesteatoma is higher in ears with congenital aural atresia (CAA) than in normally developed ears; in normal case, the ectoderm canalizes from medial to lateral to form the external auditory canal (EAC). If this process is arrested, stenosis or atresia of the EAC is a result. In such developmental disorders, when tympanic membrane is not formed, ectodermal epithelium is lost next to tympanic cavity. This lost ectodermal epithelium may initiate the growth of cholesteatoma [3].

We report a very strange case of unilateral congenital aural atresia who presented with acute mastoiditis with congenital cholesteatoma of the infratemporal fossa.

## Case presentation

**Figure 1:**
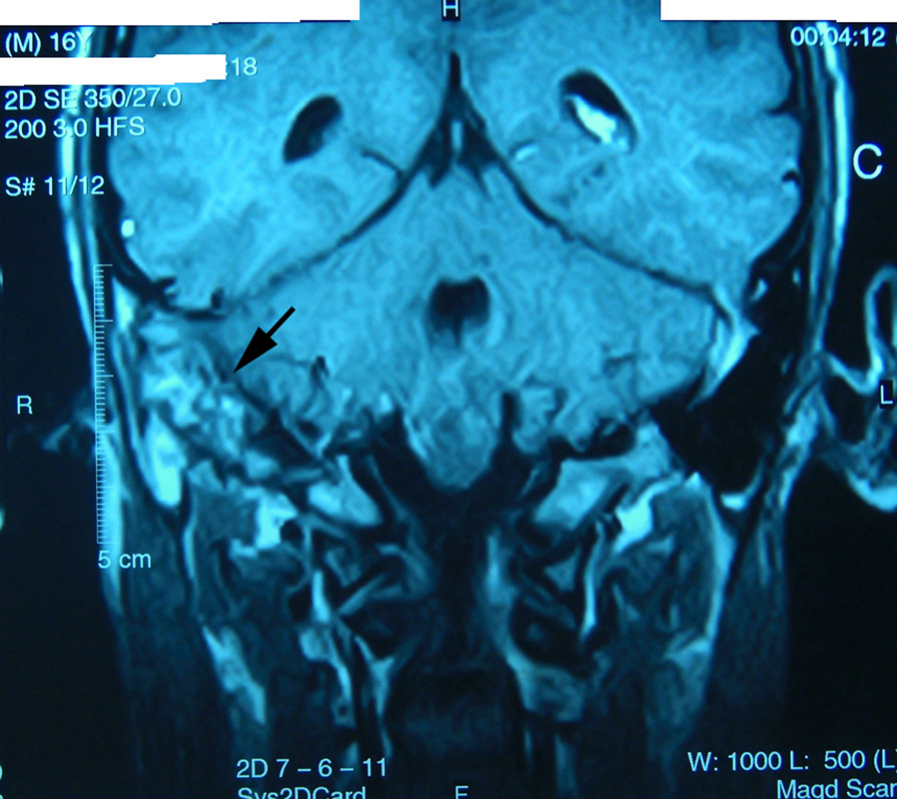
MRI of the patient with the arrow points to a large soft tissue mass in the right middle ear and infratemporal fossa.

**Figure 2:**
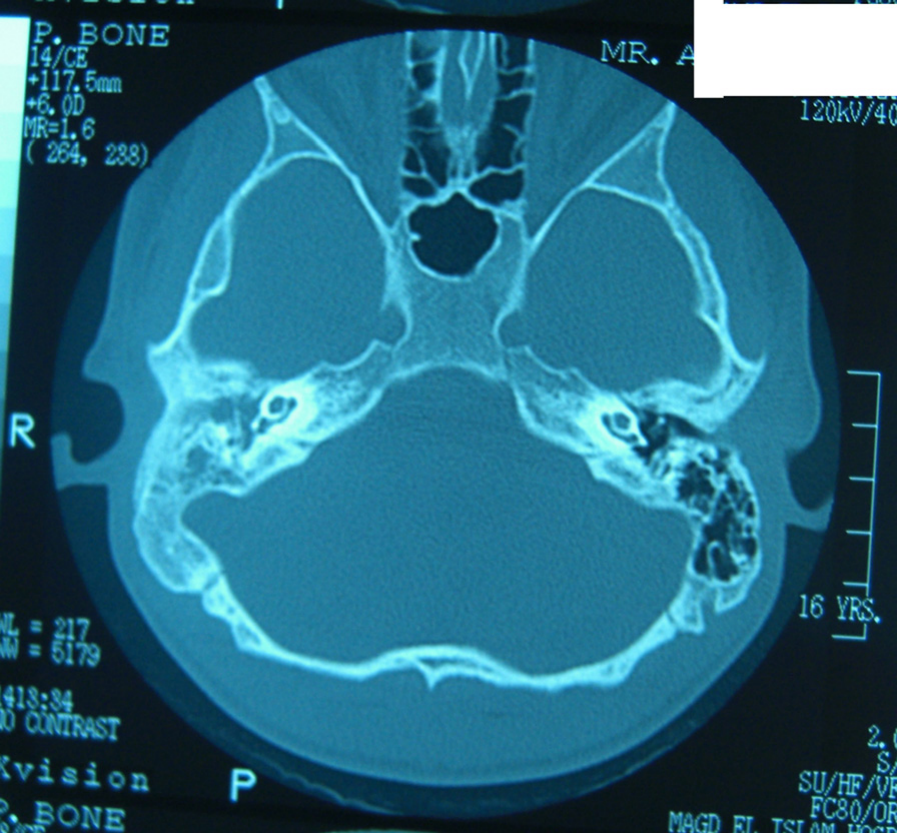
CT of the patient shows opacity of right mastoid and middle ear space.

**Figure 3:**
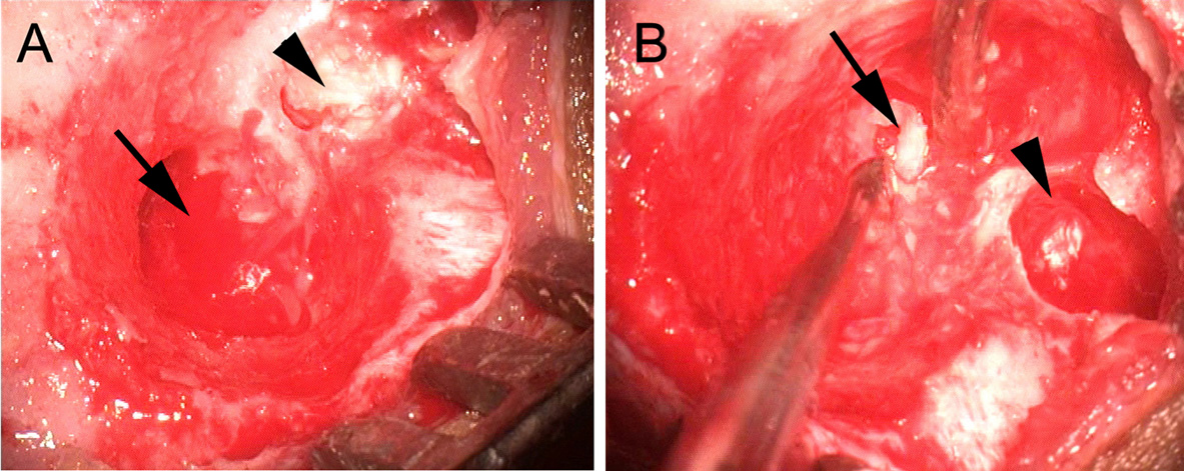
**Findings at surgery.A,** the arrow points to the newly created external auditory canal while the arrow head points to the mass in the infratemporal fossa. **B,** the arrow points to the part of the mass within the middle ear with eroded bone while the arrow head points to the cavity that has been caused by the mass.

A sixteen year-old male patient referred to our institute with post-auricular discharging sinus on his right side associated with swelling and redness around it, examination revealed that the patient had aural atresia dating since birth and microtia with acute mastoiditis that led to sinus formation on the right side, the left ear was completely normal. Two weeks before presentation, magnetic resonance imaging (MRI) was done in a private hospital due to the patient has complained of pain in right side of his head and face, it showed a soft tissue mass in the right infratemporal fossa (Figures 1), surgical excision was advised but the parents of the patient have refused at that time. On presentation, Computed tomography (CT) was done for the patient as a routine radiologic evaluation for mastoiditis and it proved the diagnosis (Figures 2). Pure tone audiometry showed conductive hearing loss with an air-bone gap of 60 dB on the right side and normal hearing of left ear. Surgical interference was carried out that had been started with post-auricular incision and excision of the unhealthy skin around the sinus, then cortical mastoidectomy was done, after identification of the lateral semicircular canal; the middle ear was entered via an atticoantrostomy approach, a soft tissue mass was seen filling the middle ear cavity, the mass was keratinous and eroding the hypotympanum and extended to outside the middle ear space. On retraction of the auricle antero-inferiorly, the large keratinous mass appeared in the infratemporal fossa that was anterior to the tempromandibular joint and inferior to the zygomatic arch which was eroded partially (Figures 3). On manipulations of the middle ear mass, we noticed movement of the infratemporal fossa mass denoting that both were one mass. The mass was removed completely with no need to widen the exposure. After removal of the mass, no ossicles were found except the stapes, also there was erosion in the floor and anterior bony wall of the middle ear space with exposure of the fibrous capsule of the tempromandibular joint, the defect was about 2 mm in diameter. Reconstruction of the external auditory canal was done through removal of the bone lateral to the middle ear space with meatoplasty of the cartilaginous portion, then a temporalis fascia graft that has been harvested early was used to create the tympanic membrane; it was placed on the head of the stapes. A split thickness skin graft that has been harvested from the right thigh was used to line the newly created external auditory canal and was sutured to edges of newly created meatus. The wound had been closed in the usual fashion with post-auricular drain and packing the newly created external auditory canal. No intra-operative or post-operative complications were reported, the pack was removed in the 10^th^ post-operative day and re-packing of the external canal was done weekly for six weeks. Histopathological examination of the mass confirmed the diagnosis of cholesteatoma, also to confirm the existence of squamous epithelial cells; immunohistochemical staining with involucrin was performed on paraffin-embedded sections of the mass, using the avidin-biotin-peroxidase complex immunoperoxidase technique [4], involucrin positivity was seen. Follow-up of the patient for 2 years and 3 months showed a patent, infection free external auditory canal with an air-bone gap of 35db. One year after the operation; MRI was done and it showed no residual or recurrent cholesteatoma.

## Discussion

Congenital cholesteatoma is a very rare disease; however it could be expected in the presence of congenital anomalies of the external ear, however it is more frequent in cases of EAC stenosis rather than in cases of complete atresia [3]. Levenson et al. [5] have suggested strict criteria for diagnosis of CC which are a whitish mass present in the middle ear cavity with normal tympanic membrane, the pars flaccida and pars tensa of tympanic membrane showed normal findings, no history of otorrhea or perforation, no history of otological surgery, history of otitid media is not an exclusion for the disease, and intramembranous cholesteatoma or giant cholesteatoma may be encountered in cases with CAA. According to the last criterion, our case has a giant temporal bone congenital cholesteatoma. In 2006, Caughey et al [6] have reported a case of CAA with congenital cholesteatoma medial to the atretic plate, with no ossicular erosion. The authors have removed the malleus-incus complex to facilitate complete removal of cholesteatoma and the conductive hearing mechanism was reconstructed using partial ossicular replacement prosthesis. However, our case showed no malleus-incus complex that raises the suggestion of its erosion by cholesteatoma and then we placed the temporalis fascia on the head of the stapes for hearing reconstruction and we succeeded to narrow the air-bone gap from 60 to 35 dB.

Regarding the site of occurrence of CC, it is usually located intradurally; less frequently (20%), an extradural location is noted. There are five general sites of extradural occurrence: the middle ear, external auditory meatus, the mastoid, squamous portion of temporal bone and the petrous apex [1]. In 2010, Chen et al [7] have reported the first case of CC of the infratemporal fossa in a five year-old child who has presented with middle ear effusion with a whitish mass behind the antero-inferior quadrant of the tympanic membrane. Computed tomography and magnetic resonance imaging has showed that the mass was present in the hypotympanum and infratemporal fossa. They concluded that the infratemporal fossa is the site of origin with limited extension to the hypotympanum; depending on the main bulk of the mass was detected in the infratemporal fossa radiologically and on intra-operative finding. However in our case, the mass was detected in the antero-inferior part of the hypotympanum medial to the atretic plate with erosion of the middle ear floor antero-inferiorly and extension of cholesteatoma mass to the infratemporal fossa. The primary site of origin of CC –in our opinion- may be the antero-inferior part of the middle ear space, as it is logic to suggest that entrapment of epithelial cell rests behind the atretic plate resulting in formation of cholesteatom that consequently eroded the bone and expanded freely within the soft tissues of the infratemporal fossa, this may explain the presence of the largest part of the mass in the infratemporal fossa rather than in the temporal bone as expansion within yielding tissues is more easier than expansion within the bone. An exposed intact fibrous capsule of the tempromandibular joint through a small bony defect was detected intra-operatively, it needed no intervention as the capsule was not herniated even with passive movement of the joint.

Acute mastoiditis has been reported to be the first presentation of CC in a normally developed ear; Hidaka et al [2] has reported a case of CC of mastoid region in a sixty five-year-old man that presented with acute mastoiditis as the first presentation.

Also, CAA may be presented with acute mastoiditis; Zalzal [8] has published a case of CAA that was presented with acute mastioditis and lateral sinus thrombosis in a two-year-old boy.

Pawełczyk and Czarski [9] published a case of CC of the middle ear in a twelve-year old child with CAA complicated by mastoid abscess. To the best of our knowledge, our case is the second case of CC with CAA to present with acute mastoiditis after the case of Pawełczyk and Czarski [9] and it is the first case to be presented with CC extended to the infratemporal fossa with the presence of CAA and mastoiditis.

## Conclusions

Finally, we conclude that CAA may be associated with CC that may extend outside the temporal bone reaching to the infratemporal fossa, also it may cause complications like mastoiditis. To avoid complications, radiologic evaluation of children with CAA is highly important for early detection of CC. Such cases should be managed surgically to drain infection and to remove the cholesteatoma mass, also atresia repair can be done successfully in the same setting.

## Consent

Written informed consent was obtained from the parents of the patient for publication of this case and accompanying images. A copy of the written consent is available for review by the Editor-in-Chief of this journal.

## Abbreviations

CC, Congenital cholesteatoma; CAA, Congenital aural atresia; EAC, External auditory canal; MRI, magnetic resonance image; CT, Computerized tomography.

## Competing interests

The author declares that he has no competing interests.

## Author’s contribution

This author is the sole author for this work.

## Author information

The study was carried out at the Department of Otolaryngology of Cairo Univesity.

The study has been funded by the author with no financial conflict of interest.

It is an original article, it has not been published before, and it is not considered for publication elsewhere.

## Pre-publication history

The pre-publication history for this paper can be accessed here:

http://www.biomedcentral.com/1472-6815/12/6/prepub
